# Medical Therapies for Heart Failure in Hypoplastic Left Heart Syndrome

**DOI:** 10.3390/jcdd9050152

**Published:** 2022-05-12

**Authors:** Angela N. Baybayon-Grandgeorge, Ashley E. Pietra, Shelley D. Miyamoto, Anastacia M. Garcia

**Affiliations:** 1 Department of Medicine, Division of Cardiology, University of Colorado Anschutz Medical Campus, Aurora, CO 80045, USA; angela.baybayon-grandgeorge@cuanschutz.edu; 2 Department of Pediatrics, Section of Cardiology, University of Colorado Anschutz Medical Campus, Children’s Hospital Colorado, Aurora, CO 80045, USA; ashley.pietra@cuanschutz.edu

**Keywords:** congenital heart disease, heart failure, hypoplastic left heart syndrome

## Abstract

Significant surgical and medical advances over the past several decades have resulted in a growing number of infants and children surviving with hypoplastic left heart syndrome (HLHS) and other congenital heart defects associated with a single systemic right ventricle (RV). However, cardiac dysfunction and ultimately heart failure (HF) remain the most common cause of death and indication for transplantation in this population. Moreover, while early recognition and treatment of single ventricle-related complications are essential to improving outcomes, there are no proven therapeutic strategies for single systemic RV HF in the pediatric population. Importantly, prototypical adult HF therapies have been relatively ineffective in mitigating the need for cardiac transplantation in HLHS, likely due to several unique attributes of the failing HLHS myocardium. Here, we discuss the most commonly used medical therapies for the treatment of HF symptoms in HLHS and other single systemic RV patients. Additionally, we provide an overview of potential novel therapies for systemic ventricular failure in the HLHS and related populations based on fundamental science, pre-clinical, clinical, and observational studies in the current literature.

## 1. Introduction

Congenital heart disease (CHD) is one of the most common birth defects worldwide, occurring in 1–2% of live births and more so in stillborn spontaneous miscarriages [1,2]. Hypoplastic left heart syndrome (HLHS) is the most common form of severe CHD. HLHS is characterized by severe underdevelopment of left sided heart structures including the mitral valve, left ventricle and aortic valve, resulting in a univentricular circulation. While single ventricle heart disease (SV) can be of either right ventricular (RV) morphology as in HLHS, or left ventricular (LV) morphology as in tricuspid or pulmonary atresia, patients with a systemic RV represent the most common SV sub-type with a prevalence of 2 to 3 per 10,000 live births worldwide [3,4,5]. In the United States alone, an estimated 1 in every 3841 infants is born with HLHS [6]. Additionally, patients with HLHS tend to have worse long-term outcomes than patients with SV of LV morphology [7,8,9]. There are inherent differences in RV and LV anatomy and physiology that may make the RV vulnerable to failure when functioning as the systemic ventricle. Anatomically, the RV lacks the middle layer of circumferential fibers which are the main source of force generation in LV contraction; therefore, the RV is limited to reliance on only longitudinal shortening for contractility [10]. Physiologically, the RV is energetically efficient when pumping to the low resistance pulmonary circulation, but is highly load-dependent and declines in performance when exposed to increased afterload [11,12]. Additionally, while the RV can adapt over time to increased afterload, there are differences in stress-related molecular adaptations in the RV compared to the LV which may also play a role in systemic RV failure [13].

Without medical intervention, HLHS is responsible for 25–40% of all neonatal cardiac deaths [14]. The significant advancements of surgical palliation and cardiac transplantation have improved the 5-, 10-, and 15-year survival of patients by nearly 40% [3,15,16,17]. However, these interventions are not curative and life-long complications including pathological cardiac remodeling and progression to heart failure (HF) are still important consequences of HLHS [18,19]. In general, early recognition and treatment of HLHS-related complications are essential to improving patient outcomes. However, there are no Class I, Level A evidence-based treatments for HF associated with any form of CHD, and management of HF in patients with HLHS is particularly challenging due to their unique anatomy and physiology.

**Figure 1:**
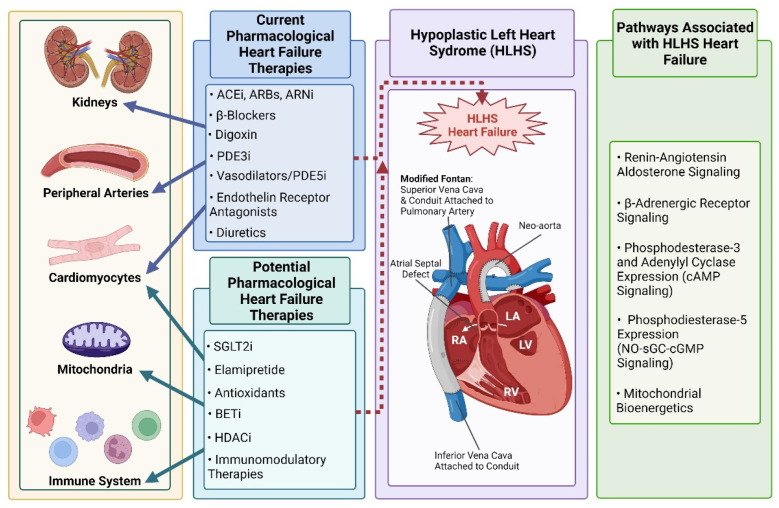
Current pharmacological therapies and potential novel therapeutic targets that could modulate Hypoplastic Left Heart Syndrome (HLHS)—associated heart failure (HF). Current commonly used adult HF therapies have demonstrated mixed or inconclusive effects on HF risk and patient outcomes in the HLHS population. Targets implicated in HLHS HF that could be amenable to pharmacological treatment and improve outcomes for HLHS patients are summarized. Created with BioRender.com (accessed on 24 April 2022).

While the discussion thus far and throughout this review is on HLHS, there are other CHDs resulting in a single systemic RV circulation such as right-dominant atrioventricular septal defect, and some forms of double outlet and double inlet right ventricle. For the purposes of this review, while HLHS serves as the prototypical CHD, the discussion can be extrapolated to any CHD with a single systemic RV. There are many etiologies for HF in patients with HLHS including neo-aortic arch obstruction, valvular insufficiency, a restrictive atrial septum, arrhythmias, and obstruction within the Glenn or Fontan circulation. Here, we review the most commonly used medical therapies for *myocardial failure* in single systemic RV HF as well as potential novel therapies for systemic ventricular failure in HLHS and related CHD populations (Figure 1).

## 2. Current HLHS Heart Failure Therapies

For many reasons, there are a limited number of prospective clinical studies in children with CHD. Children represent a vulnerable population and the number of patients with any given type of CHD is fairly small, increasing the chances that a clinical trial will be dramatically underpowered. As a consequence, most of the current HF treatments used in the pediatric CHD population have their basis in adult HF research [20]. However, given the unique age, anatomical, and physiological differences in these populations, it is unsurprising that many of the evidence-based adult LV HF therapies fail to show benefit for SV HF [19]. Reviewed below are the most commonly applied current therapeutic interventions for the treatment of HLHS HF.

### 2.1. ACE Inhibitors, ARBs, and ARNis

Angiotensin-converting enzyme inhibitor (ACEi) therapy has been traditionally used in high-risk adult HF patients to delay pathological ventricular remodeling by blocking the Renin-Angiotensin Aldosterone System (RAAS) [21]. Physiological upregulation of RAAS has been demonstrated in the HLHS population, suggesting that ACEi use in HLHS patients equivalates a similar response to that seen in adults, in addition to decreasing afterload on the systemic ventricle [22]. A double-blind, randomized study of the ACEi, *Enalapril*, in infants with SV who did not have HF failed to demonstrate any benefit in somatic growth, ventricular function, or HF prevention [23]. Based on this study, prophylactic use of ACEi in SV is not recommended. A separate study looking at the Angiotensin Receptor Blocker (ARB), valsartan, in adult SV patients with single RV dysfunction, demonstrated no beneficial impact on systemic RV ejection fraction, maximum exercise capacity, quality of life, or clinical outcomes after 3 years of continuous therapy [24]. Further subgroup analysis of this study, however, demonstrated that after the three-year follow up, RV ejection fraction in the valsartan group remained unchanged, while in the placebo group RV ejection fraction was significantly reduced. There have been no controlled studies of angiotensin receptor-neprilysin inhibitors (ARNi) in the HLHS population and small descriptive series have had mixed results [25,26]. Confounding variables such as age, ventricular morphology, presence of ventricular dysfunction, valve regurgitation, HF symptoms, and renal dysfunction should be considered prior to initiating therapy with ACEi or ARBs in the HLHS population.

### 2.2. β-Blockers

β-Adrenergic receptor antagonists (β-Blockers), such as carvedilol and metoprolol, are fundamental in the standard care of adult HF patients [27]. However, despite their association with dose-related LV function improvement, reverse-remodeling, and reductions in mortality in adult patients, no such efficacy has been found with β-Blocker therapy in the SV HF population. While a handful of small retrospective case reports and studies have shown beneficial effects for SV HF with β-Blocker therapy [28,29,30], a randomized controlled clinical trial in children with systolic HF comparing placebo to the non-selective β-Blocker, carvedilol, demonstrated no benefit for SV patients. In fact, patients with a systemic RV tended to have worse outcomes in response to carvedilol [20]. Moreover, it has been shown that β-Adrenergic receptor adaptations are uniquely altered in the failing HLHS hearts compared to failing adult hearts, providing a potential explanation for the lack of efficacy of β-Blockers in those with a systemic RV [31,32].

### 2.3. Digoxin

Digoxin is a cardiac glycoside, derived from the purple foxglove flower, and was one of the first drugs used for the treatment of HF and arrhythmias. However, due to multiple drug interactions and the overlap in therapeutic and toxic concentrations that requires close monitoring, digoxin is now only occasionally used and is no longer considered a first-line therapy [33]. These limitations are not as problematic in the pediatric population that have fewer comorbidities and concomitant medications [34,35]. A retrospective study of infants with HLHS in the National Pediatric Cardiology Quality Improvement Collaborative (NPCQIC) database demonstrated improved survival for those infants discharged home on digoxin after the Norwood procedure [36]. However, there have not been prospective controlled trials of the use of digoxin for the treatment of SV HF.

### 2.4. PDE3 Inhibitors

While the chronic use of phosphodiesterase-3-inhibitors (PDE3i), such as milrinone, is associated with increased mortality in adults with HF [37,38], this class of drugs is commonly used in children. Milrinone for example, has been shown to prevent post-operative low cardiac output syndrome in children with CHD [39]. In addition, it has demonstrated lusitropic, and systemic and pulmonary arterial vasodilatory effects, and has been shown to augment renal blood flow [40,41,42]. A recent study demonstrated improved one-year survival in infants with SV receiving perioperative treatment with milrinone as opposed to epinephrine and dopamine after the Norwood procedure [43]. Milrinone has been shown to improve HF symptoms in HLHS patients, including reducing frequency of HF-related emergency department visits and hospital admissions [44]. In addition, PDE3i therapy in pediatric SV HF is commonly used as a bridge to transplant [31]. PDE3 is a certified regulator of myocardial contractile function via cyclic-AMP (cAMP) signaling and its modulation of phospholamban (PLN) and sarcoplasmic reticulum calcium-ATPase (SERCA) [45]. Preservation of PLN activity in the myocardium and distinct cAMP compartmentalization in children with SV HF, may account for the beneficial response to PDE3i therapy observed between SV and adult HF patients [46].

### 2.5. Vasodilators

In the HLHS population, maintaining a low pulmonary vascular resistance is important for optimal circulation, particularly after Fontan palliation when blood flow to the lungs is entirely passive. As a consequence of this unique physiology, various pulmonary vasodilator therapies including endothelin receptor antagonists, prostanoids, and phosphodiesterase-5-inhibitors (PDE5i) have been used in the treatment of HLHS HF.

#### 2.5.1. Endothelin Receptor Antagonists

Circulating concentrations of a vasoconstrictor, plasma endothelin-1, are increased in post-Fontan HLHS patients [47]. Several studies conducted in post-Fontan patients treated with the endothelin rector antagonist, bosentan, observed improved exercise capacity and cardiac performance without significant adverse events including hepatotoxicity [48,49,50]. Despite these encouraging results, this class of drugs is not commonly used in the treatment of SV HF. Additional studies are required to better assess the long-term effects of endothelin receptor antagonism therapy, such as that on Fontan-associated liver disease.

#### 2.5.2. Prostanoids

Prostanoid, E-type 1 prostaglandin (PGE1), is commonly used in HLHS neonates prior to Norwood palliation to maintain patency of the ductus arteriosus [51,52,53]. HLHS patients are dependent on a patent ductus arteriosus to provide systemic blood flow, due to severe obstruction of the left ventricular outflow tract. PGE1 dilates the ductus arteriosus as a postnatal therapeutic to maintain the right to left shunting of systemic blood flow prior to surgical intervention. Prostanoid use in older SV patients, particularly post-Fontan is rare; however, several small studies have looked at inhaled iloprost in patients with Fontan circulation and found improvement in peak oxygen pulse and maximum VO2, as well as improved exercise capacity and cardiac output [52,53].

#### 2.5.3. PDE5 Inhibitors

PDE5 inhibitors (PDE5i) are often used in the HLHS population to treat pulmonary hypertension and improve pulmonary blood flow as reviewed in [54]. PDE5 is a cyclic-GMP (cGMP) specific PDE, and PDE5 expression is upregulated during increased cardiac stress and is associated with unfavorable myocardial responses [55,56,57]. However, while PDE5i use as an adult HF therapeutic is inconsistent [58,59], a recent multicenter, longitudinal, phase III clinical trial of udenafil therapy in post-Fontan SV adolescents demonstrated improvements on measures of exercise performance [60]. While the primary target of PDE5i therapy in SV is the pulmonary vasculature, it has been shown that PDE5 is expressed in the failing myocardium of HLHS patients, suggesting PDE5i may have therapeutic effects on the myocardium directly. Additionally, recent pre-clinical studies have demonstrated improved mitochondrial function in response to PDE5i therapy in the heart and liver, and in diabetic adipocytes [61,62,63]. Thus, while further research is needed to demonstrate the most appropriate indications and mechanisms involved in the beneficial effects of PDE5i use in SV patients, its current association with improved exercise tolerance, pulmonary blood flow, and systemic RV function is promising [57,60,64].

#### 2.5.4. Guanylate Cyclase Inhibitors

Riociguat, a novel soluble guanylate cyclase (sGC) inhibitor, was recently investigated for the treatment of pulmonary hypertension associated with CHD [65,66]. Riociquant acts by both sensitizing sGC to endogenous nitric oxide (NO) and by directly stimulating sGC independently of NO. Similar to PDE5i therapy, sGCi therapy restores the NO-sGC-cGMP pathway, thereby increasing cGMP and stimulating vasodilation. The efficacy and safety of riociguat in a subset of patients with persistent/recurrent pulmonary arterial hypertension after correction of CHD were extrapolated from a large randomized, double blind, placebo-controlled phase III trial of riociguat in patients with pulmonary arterial hypertension [67]. Riociguat was well tolerated in CHD patients and improved 6-min walk distance, pulmonary vascular resistance, N-terminal of the prohormone of brain natriuretic peptide (NT-proBNP) levels, and WHO functional class. However, while this study suggests potential benefit of sGCi therapy in those with CHD there were no patients with HLHS specifically included in the study.

### 2.6. Diuretics

Congestive symptoms (edema, dyspnea, orthopnea) are a hallmark of HF and are secondary to increased extracellular volume and increased ventricular filling pressures. Diuretic therapy aims to mitigate the consequences of this volume expansion and is therefore commonly used for symptom management in patients with HF [68]. Interestingly however, evidence to support the use of diuretics, even in adult HF populations, is lacking. There are several types of diuretics including loop diuretics (e.g., furosemide), thiazides (e.g., hydrochlorothiazide), osmotic agents (e.g., mannitol), carbonic anhydrase inhibitors (e.g., acetazolamide), and potassium-sparing (e.g., spironolactone). Loop diuretics are the most commonly prescribed as they are generally efficacious and cost-effective. Thiazide diuretics are often employed when patients become resistant to loop diuretics. While the use of diuretics is reasonable in patients with HLHS and congestive HF symptoms, once symptoms improve and euvolemia is achieved attempts at discontinuing or decreasing diuretic use can limit complications such as electrolyte derangements, worsening renal function, and fractures in small children [69].

## 3. Potential Future HLHS Medical Therapies

Despite advances in medical and device therapy for the treatment of HF, morbidity and mortality remain high [70]. Therefore, there is a continual interest in identification of new therapies for the treatment of adult LV or RV HF, which have the potential to subsequently benefit children with CHD, including those with HLHS.

### 3.1. Mitochondrial Targeted Therapies

Although the pathophysiology of HF is complex, mitochondrial dysfunction is common across various HF etiologies [71], including HLHS [72,73,74]. Mitochondrial abnormalities include impaired mitochondrial electron transport chain (ETC) activity, increased formation of reactive oxygen species (ROS), altered metabolic substrate utilization, and aberrant mitochondrial dynamics, resulting in reduced capacity to generate myocardial adenosine trinucleotide phosphate adequate (ATP) for appropriate myocardial function reviewed in [71]. Therefore, mitochondria represent an important target for SV HF therapy with considerable potential to improve cardiac function.

#### 3.1.1. Elamipretide (Bendavia, MTP-131, SS-31)

Cardiolipin is a mitochondrial phospholipid critical in facilitating normal ATP generation by anchoring proteins of the ETC onto the inner mitochondrial membrane [75]. Elamipretide (also referred to as Bendavia, MTP-131, and SS-31) is a novel aromatic-cationic, cell-permeable tetrapeptide that targets mitochondrial cardiolipin to help enhance mitochondrial function. Several pre-clinical studies have demonstrated that SS-31 significantly improved LV function, increased myocardial ATP synthesis, and prevented pathological LV remodeling in animal models [76,77,78]. However, clinical studies evaluating the effects of SS-31 in patients with HF have yet to report significant improvement on LV systolic or diastolic function [79,80]. This may be due to many variables, one of which is the short-term SS-31 therapy in clinical studies versus the higher concentrations and more chronic exposures in pre-clinical studies. Specific to the SV population, there is evidence of dysregulation of the biosynthesis and remodeling of the mitochondrial phospholipid cardiolipin in failing SV hearts that could have implications for mitochondrial function [81]. An ex vivo study utilizing explanted pediatric and adult cardiac specimens (including hearts from SV patients) demonstrated that acute SS-31 treatment improved cardiac mitochondrial function [78], further suggesting the potential benefit of mitochondrial targeted therapies for the treatment of SV HF.

#### 3.1.2. Antioxidants

Over the past several years, antioxidants have gained increased popularity for the treatment of several disease etiologies. In fact, several of these molecules are undergoing investigations for the treatment of adult HF for their antioxidant properties and ability to stimulate mitochondrial biogenesis. In a small clinical trial, resveratrol, a naturally occurring antioxidant, improved LV function, endothelial function, lowered cholesterol levels, and protected against unfavorable hemorheological changes in patients with coronary artery disease [82]. Other antioxidant molecules such as oxypurinol, allopurinol, sapropterin, nicotinamide adenine dinucleotide (NAD+), vitamin E, and folic acid have been shown to protect the heart from pathological remodeling and improve cardiac function in various pre-clinical studies [reviewed in 83,84]. Moreover, rodent models overexpressing antioxidant enzymes such as superoxide dismutase (SOD), a catalase, also show significant benefit in terms of cardiac remodeling and function in the setting of induced cardiac stress. However, in the clinical setting, antioxidant therapies have yielded varied and somewhat disappointing results [83,84,85]. Perhaps the subcellular compartmentalization of ROS formation may explain the lack of efficacy of untargeted antioxidant therapy. Antioxidants that selectively accumulate in the mitochondrial matrix, such as mitoquinone (MitoQ) may prove to be more efficacious in the setting of HF. For example, while MitoQ has not yet been evaluated in HF patients, dietary supplementation with MitoQ improved endothelial function in elderly individuals [86] and acute oral therapy improved flow-mediated vasodilation, walking capacity, and time to claudication in patients with peripheral artery disease [86]. Whether these therapeutic strategies will improve outcome in specific subsets of HF patients, including those with HLHS, is yet to be determined, but deserves further consideration.

### 3.2. SGLT2 Inhibitors

Sodium-glucose co-transporter-2 (SGLT2) inhibitors represent a recent adult HF therapeutic directed at glucose metabolism. SGLT2 proteins are expressed in the proximal convoluted tubule of the kidneys and are responsible for the majority of the reabsorption of glucose filtered by the kidneys [87]. SGLT2 inhibition (SGLT2i), therefore, blocks the reabsorption of filtered glucose in the kidneys in an insulin-independent mechanism, resulting in an increased amount of glucose secreted in the urine. Many clinical trials have confirmed the efficacy SGLT2i in lowering glycemic levels in the type 2 diabetes (T2D) population [88,89], and large-scale clinical trials present evidence of the efficacy of SGLT2i treatment in preventing HF in patients with T2D [89,90]. In a systematic meta-analysis, SGLT2i also appear to significantly reduce systolic and diastolic blood pressure [91]. Additionally, a second meta-analysis suggests reduced cardiovascular mortality and HF hospitalizations among adult HF patients in multiple cardiovascular outcome trials, even in the absence of T2D reviewed in [92]. Interestingly, the cardiac benefits seen in response to SGLT2i therapy might be partly explained by their effects on ion handling and metabolism of cardiac myocytes reviewed in [84]. In light of the evidence of SGLT2i’s efficacy in substantially improving outcomes for adult patients with HF with reduced ejection fraction (HFrEF), the translation of this class of medication to treat HLHS HF deserves further investigation.

### 3.3. BET Inhibitors

Bromodomain and extraterminal motif (BET) inhibitors are a class of drugs that have been proposed as a therapeutic for multiple cancer types, and more recently, for the treatment of pulmonary arterial hypertension [93,94]. BET proteins contain two N-terminal domains that bind to an acetylated lysine residue on histone tails, playing a more indirect role in initiating and continuing transcription and regulating the cell cycle. Though further investigation is required, one pre-clinical study found that rodent models exposed to chronic hypoxia showed significant RV hypertrophy, increased systolic pressure and contraction speed compared to control, that was reversed with BET inhibitor (I-BET151) treatment [95]. This study provides promising evidence of the potential beneficial effects of BET inhibition on pulmonary hypertension, which could subsequently translate to patients with Fontan physiology [94,96].

### 3.4. HDAC Inhibitors

Histone deacetylases (HDACs) are one class of enzymes that remove the acetyl group from lysine residues of both histone and non-histone proteins and represent yet another novel potential therapeutic [97]. A study utilizing explanted cardiac specimens, showed increased activity and expression of class I, IIa, and IIb of HDACs in HLHS myocardium compared to normal controls [98]. A similar effect showing elevated HDAC catalytic activity and protein expression was seen in a neonatal rodent model of hypoxia-induced RV hypertrophy, suggesting that targeting HDACs may be a promising novel therapeutic for systemic RV failure in the HLHS population.

### 3.5. Immunomodulatory Therapies

Mounting evidence suggests that the immune system contributes significantly to cardiac function and plays a role in the pathophysiology of HF. Specific depletion of cardiac tissue-resident macrophages for example, impairs clearance of damaged mitochondria in the myocardium, leading to metabolic alterations and ultimately ventricular dysfunction [99,100]. Moreover, circulating peripheral blood mononuclear cells are a source of pro-inflammatory cytokines in the failing adult heart [101,102]. Importantly, neonatal thymectomy is routinely performed in infants with HLHS to gain better visualization during surgical palliation, and while the consequences are not well understood, evidence suggests altered immune cell composition and impaired immune cell-mediated responses in these patients [103,104,105]. Therefore, a thymectomy may significantly impact the formation of the immune cell pool, which in turn can influence the development of clinically-significant co-morbidities, including the progression to HF in HLHS patients. Therefore, immunomodulatory therapies may represent another potential novel approach to treatment and deserve further consideration in this population.

## 4. Conclusions

Altogether, there are many factors contributing to the lack of evidence-based therapies for the treatment of HF in HLHS. Due to the relative rarity of HLHS, it is not possible to perform large, randomized, placebo-controlled clinical trials, as is conducted for adult HF therapeutics. There is an evolving body of evidence, much of which is described above, that the failing HLHS myocardium bears unique attributes in comparison to the adult failing heart, suggesting some adult-based therapies may not be efficacious, while others may hold more promise. Additionally, basic science and pre-clinical investigations of the failing HLHS myocardium have identified potential myocardial therapeutic targets such as PDE5 and mitochondrial dysfunction, which deserve further study in this population. Through a combination of pre-clinical, clinical, and observational studies, advancements in the clinical care and outcome of patients with HLHS HF are possible.

## Data Availability

Not applicable.
